# Development of the Video Analysis Scale of Engagement (VASE) for people with advanced dementia

**DOI:** 10.12688/wellcomeopenres.16189.2

**Published:** 2021-05-20

**Authors:** L.L. Daniel Lai, Sebastian J. Crutch, Julian West, Emma Harding, Emilie V. Brotherhood, Rohan Takhar, Nicholas Firth, Paul M. Camic

**Affiliations:** 1 Clinical Psychology, Kwong Wah Hospital, Hospital Authority, Hong Kong, Hong Kong; 2 Dementia Research Centre, Queens Square Institute of Neurology, University College London, London, UK; 3 Open Academy, Royal Academy of Music, London, UK

**Keywords:** dementia, observational measurement, video recording, engagement, neurocognitive disorder, quality of life, wellbeing

## Abstract

**Background**: The current study sought to develop a valid, reliable and unobtrusive tablet computer-based observational measure to assess engagement of people with advanced dementia. The Video Analysis Scale of Engagement (VASE) was designed to enable the rating of moment-by-moment changes in engagement during an activity, which would be useful for both future research and current residential care.

**Methods**: An initial version of the VASE was tested. Face validity and content validity were assessed to validate an operational definition of engagement and develop an acceptable protocol for the scale. Thirty-seven non-professional and professional volunteers were recruited to view and rate level of engagement in music activities using the VASE.

**Results**: An inter-class coefficient (ICC) test gave a high level of rating agreement across professionals and non-professionals.  However, the ICC results of within-professionals were mixed. Linear mixed modelling suggested that the types of interventions (active or passive music listening), the particular intervention session being rated, time period of video and the age of raters could affect the ratings.

**Conclusions**: Results suggested that raters used the VASE in a dynamic fashion and that the measure was able to distinguish between interventions. Further investigation and adjustments are warranted for this to be considered a valid and reliable scale in the measurement of engagement of people with advanced dementia in a residential care setting.

## Introduction

Dementia is a growing challenge for nations worldwide ( Alzheimer’s Society, 2015; World Health Organisation, 2018). Non-pharmacological interventions (NPIs) are thought to be a meaningful and preferred approach to care for people living with a dementia (PWD) ( Cabrera *et al.*, 2015). These interventions include, for example, cognitive stimulation therapy ( Orrell *et al.*, 2017), singing ( Camic *et al.*, 2011), music and art therapies ( Deshmukh *et al.*, 2018; Raglio *et al.*, 2015), talking therapies ( Cheston & Ivanecka, 2017), and others. In earlier studies, the evaluation of whether these interventions were successful often relied on self-report measures or staff observations of behavioural and psychological symptom changes; factors such as social interaction and engagement had not been considered as important factors or useful outcomes in dementia care ( Du Toit *et al.*, 2019; Sung & Chang, 2005). In recent years, there has been an emphasis on promoting wellbeing for PWD through social engagement within their immediate environments ( Martyr *et al.*, 2018). Such an approach recognizes that focusing solely on emotional and behavioural outcomes to determine if an intervention is successful might pose a danger where interventions are “prescribed” to PWD based on those outcomes, without considering the individual's personal choice.

Beard, Knauss & Moyer (2009) found that people with dementia (PWD) continued to want an enriched life after being diagnosed. Having positive attitudes and engaging with physical, mental and social activities are thought to be helpful to maintain an enriched life ( Beard *et al.*, 2009). Kitwood’s (1997a) person-centered approach highlighted the importance of maintaining personhood in dementia care, stressing the need to promote social inclusion to maintain identity for the individual. The maintenance of inclusion and identity could be achieved through self-directed support in dementia care ( Mental Health Foundation, 2011), such as placing a strong emphasis on the importance to respond to needs “in the moment” ( Dunne, 2002). 

Understanding the choices and needs of people at advanced stages of dementia is not always clear as deterioration in memory, difficulties in communication and impairments in daily activities increase as the condition progresses. Consequently, being able to express basic needs and wants becomes more difficult and PWD perspectives become “lost” ( Kumar & Kuriakose, 2013; Lai, 2015). Providing appropriate care that is meaningful for the PWD relies on staff and carers’ understanding and familiarity with the individual, which is enhanced by careful observation of daily interactions ( Mental Health Foundation, 2011). 

### Engagement

One way to assist staff and carers to understand PWD’s preferences is to consider a person with dementia’s level of engagement with particular activities. Engagement, as a form of social interaction, is thought to be an important factor in determining the effectiveness of interventions in their ability to promote meaningful activity ( Jones *et al.*, 2015). One of the key aspects of person-centred care is the recognition that all human life is grounded in social relationships ( Brooker & Latham (2016)). Therefore, advanced dementia care should focus on creating a rich social environment to foster personal growth by maintaining engagement, relationships and activities appropriate to the level of impairment ( Mitchell & Agnelli, 2015). Other than human interaction, Cohen-Mansfield *et al.* (2009) suggested that engagement could refer to “the act of being occupied or involved with an external stimulus” (p. 2); this idea suggests that being engaged not only means connecting with people, but also with other stimuli, such as objects, music and activities.

Engagement within a social context is often determined by an individual’s participation in the activities of a social group ( Prohaska *et al.*, 2012). Zhang, Jiang, & Carroll (2011) suggested that engagement means that the member stays in the group and interacts with others. Perugia *et al.* (2018) defined engagement for those with dementia as wellbeing, enjoyment and active involvement triggered by meaningful activities. For the purpose of this study, engagement is conceptualized as a state of wellbeing and involvement triggered by participating in activities within a group.

During the advanced stages of dementia, when language skills often deteriorate, residential care is sometimes a consideration ( Herrmann & Gauthier, 2008). The environment of residential care can create difficulties in day-to-day social life, challenge a sense of identity, reduce self-confidence and as a result engagement in daily activities diminishes over time ( Clair *et al.*, 2005; Hubbard *et al.*, 2002). Reduced engagement can lead to boredom, loneliness and depression ( Cohen-Mansfield *et al.*, 2009); it is therefore essential for people with dementia to participate in activities that promote positive social interactions in residential care. Research further suggests that engaging in meaningful and enjoyable activities can lead to a better quality of life ( Smit *et al.*, 2016), fewer behaviour problems ( Braun, 2019) and increased positive emotions ( Kolanowski *et al.*, 2014).

It is important to note that a lower level of engagement in particular activities should not be seen as a symptom or a lack of ability, but rather, may be an indicator of having a strong sense of self ( Sabat, 2006). Individuals rejecting participation in an activity might be indicating an ability to advocate and express needs. Sabat (2006) proposed that “self” remains even in the advanced stages of dementia and there are three forms of self, each with different attributions. The most vulnerable self for people in the advanced stage of dementia is a “publicly presented persona that requires the cooperation of others”. To protect this part of the self, Sabat (2006) stressed that carers should provide good quality interactions that support relationships and the role of the individual. Sabat’s (2006) theory is a further development of Kitwood’s concept of personhood focusing on person-centred care, which identifies activities to stimulate engagement at advanced stages of dementia. As such, gauging engagement with this population becomes an important care issue.

### Methods to measure engagement

A review conducted by Curyto *et al.* (2008) concluded that observational measures are the “gold standard” in assessment for older people at the advanced stages of dementia ( Kawulich, 2012). Observation techniques have previously been used to investigate the process and interactions in dementia care ( Curyto *et al.*, 2008; Engström *et al.*, 2011; Gaugler *et al.*, 2013). Observations are thought to be able to gather meaningful data about day-to-day functioning that might be missed by standard questionnaires ( Algar *et al.*, 2016).

Limitations to the traditional observational measures include requiring observers to be present during a given session, which is costly and labour-intensive, as well as potentially creating stress that could affect group interactions ( Carthey, 2003). Less intrusive and cost-effective alternatives could address these concerns. Robert *et al.* (2010) noted that the existing observational engagement measures are difficult to use, even for professionals. Their review considered 68 available assessment scales in Alzheimer’s disease for various purposes and concluded that there was a need for an easy-to-administer scale for identifying response to therapy in daily practice ( Robert *et al.*, 2010). The existing observational protocols are time-intensive to administer and frequently unable to monitor direct carer-PWD interactions that reflects person-centred care ( Gaugler *et al.*, 2013). For example, the Observational Measurement of Engagement (OME) has been validated to examine the engagement of PWD in interventions such as music ( Cohen-Mansfield *et al.*, 2009). This follows a complex protocol requiring formal training and a substantial amount of time to learn. Its complexity potentially reduces the accessibility of such measures for clinicians and care staff. Consequently, staff carers might struggle to find appropriate measures to assess whether particular activities are engaging to people at the advanced stages of dementia. Furthermore, the available engagement measures constitute either a single score system or an average score from a certain period (e.g. 30 seconds to 1 minute). However, human interactions are “dynamic”, changing “moment-by-moment”, which this method of measuring might miss. Therefore, there is a need to create a flexible measure that allows those doing the rating (henceforth “raters”) to capture these dynamic changes as and when they observe them.

### Video-based observation

Video-based observation offers a good alternative as it was developed to be an unobtrusive method, minimising disruption to the social setting through the presence of researchers and observers. This method can also capture multiple, complex interactions simultaneously while gathering a larger amount of data than traditional observational methods ( Asan & Montague, 2014. A further benefit of using video analysis with a severely impaired dementia population is that it allows both researchers and care staff to closely view group interactions, meanwhile facilitating an examination and understanding of subtle behaviours occurring within the group ( Asan & Montague, 2014). Video analysis can also enable the use of raters from the wider social system (e.g. lay people, family carers and non-dementia experts), therefore permitting a more comprehensive perspective of care. A wider perspective of care, including family’s views and support, is thought to be important in dementia care ( Moore *et al.*, 2012).

### Music as an intervention

The rationale to use music activities as a basis for developing an observational measure was based on music being seen as a useful intervention across all stages of dementia ( Abraha *et al.*, 2017; Alzheimer’s Society, 2018). Both live and recorded music interventions have also been widely used in community and residential care settings ( Clare & Camic, 2020). These interventions have been found to increase quality of life Vasionytė & Madison, 2013), as well as promoting other physio-psychosocial outcomes in dementia ( Cooke *et al.*, 2010). Most importantly, such interventions have been found to improve levels of engagement ( Eggert *et al.*, 2015) and are therefore a suitable non-pharmacological intervention for the purpose of this study. Music activities are thought to engage by promoting relaxation or creating a sensory stimulant, which have been thought to increase alertness, reduce agitation and improve quality of life for those across different stages of dementia ( Cohen-Mansfield *et al.*, 2009; Strøm *et al.*, 2016).

Music interventions are of particular importance for people at the advanced stage of dementia, and although language ability has deteriorated, musical recognition abilities are relatively preserved ( Baird & Samson, 2015). This lends support to the theory of individualised music intervention for agitation (IMIA), which suggests that music acts as a medium for communication for those who have an impaired ability to understand verbal language ( Clare *et al.*, 2020; Gerdner, 2012). Currently, one widely used music program in the United Kingdom (UK) for advanced dementia is Music for Life (MfL) ( Music for Life, 2014). MfL is an approach that brings together professional musicians, care staff and people living with dementia through interactive music to enhance their quality of life ( Rose, 1993).

### Aim and objectives

An effective and easy-to-administer measure that assesses engagement in advanced dementia has yet to be developed. As part of the Created Out of Mind project ( Brotherhood *et al.*, 2017) the present study aimed to develop a non-intrusive, easily used video-based observational tool to assess level of engagement during a music intervention (MfL). The development of the measure, the Video Analysis Scale of Engagement (VASE), was carried out in two stages: the protocol development stage and the validation stage. This paper presents the development of the VASE and reports its psychometric properties in terms of validity and reliability analyses. The research question, objectives and hypotheses of the study are presented in Box 1.

Box 1. **Project objectives and hypotheses**

**Primary research question**

Can an observational rating tool effectively measure the engagement of people with advanced dementia in a music activity?

**Overall objectives**

To develop an observational rating tool with a user-friendly operational protocol to measure the level of engagement of people with advanced dementia. The intervention used for observing the participants’ level of engagement is a music-based activity (Music for Life, MfL) in residential care.

***Objective 1.*** Identify an operational definition of engagement for the Video Analysis Scale of Engagement (VASE) and determine its face and content validity.

***Objective 2.*** Determine inter-rater reliability and intra-rater reliability of VASE by comparing groups of raters (non-professionals and dementia care professionals). If the scale is found to be reliable, assess the validity of the scale to differentiate engagement by comparing two conditions, namely the MfL and passive listening (PL) groups.

***Objective 3.*** Assess whether other variables, including age, gender, ability to play a musical instrument, participation in a choir in the past or the present, types of session (MfL versus PL) or the order of videos raters rated (order effect), might affect the engagement score.

**Hypotheses**

***Hypothesis 1.*** Related to objective 2 above, there will be a significant correlation between the VASE rating by dementia experts and non-professionals.

***Hypothesis 2.*** Related to objective 2, there will be a correlation between the VASE rating among the dementia experts as well as that among the non-professionals.

***Hypothesis 3.*** Related to objective 2, the rating tool will be sufficiently sensitive to differentiate the engagement level between the two music-based activities (MfL versus PL).

***Hypothesis 4.*** Related to objective 3, extraneous variables will not affect the rating.

## Methods

### Design and procedure

This study adopted a mixed-methods design ( Creswell *et al.*, 2003) and was separated into development and experimental stages. In the development stage a qualitative method was used to identify an operational definition of engagement, followed by developing a protocol for the VASE. Face validity and content validity were then assessed. Face validity is the appropriateness, sensibility, or relevance of the tool and its items as they appear to the persons answering the test ( Holden, 2010), whereas content validity investigates whether the scale adequately covers the content that it should, with respect to the variable (i.e. engagement) ( Heale & Twycross, 2015). The experimental stage followed a quasi-experimental design to investigate the detection and rating of different levels of engagement in an active group (MfL) and a passive listening (PL) group. The reliability of the VASE was then assessed. Criterion validity was considered to compare how well the VASE measured engagement against another validated measure ( Heale & Twycross, 2015). This validity test was not carried out for two reasons. Firstly, there were no tools available that measured moment-by-moment level of engagement. Secondly, if the VASE was to compare with other “non” moment-by-moment engagement tools, such as OME, participants would have to pause the VASE rating every 15 seconds to complete another rating, making the duration of the experiment much longer, more complex and more difficult for participants to focus on watching and rating the video itself. 

### Measures

***Video analysis scale of engagement (VASE).*** The VASE is an offline application written in the hypertext markup language (HTML) ( Lai *et al.*, 2020b) and developed as a computer tablet-based program consisting of three main parts: 1) a 500 mm × 400 mm viewing box that allows raters to view a preloaded video; 2) a seven-point Likert type scale (from 0 (low engagement) to 7 (high engagement) to record changes in engagement while viewing the video; and 3) an exact time stamp to determine time of rating relative to the start of each video. VASE adopts a continuous scoring system using a seven-point Likert-type scale to assess level of engagement whilst viewing a video segment. Responses and time of the responses are automatically recorded by the software. The VASE enables raters to respond in real time by simply tapping a scale on the screen without the need to stop the footage to record ratings. The frequency of rating was dependent upon the individual rater’s decision and judgement with each rating captured to the nearest second. This occurred for each second of scoring, whether the rater had changed the score or the score remained the same. All participants would, therefore, have a total of 1200 seconds of score, which is the total length of the video. Thus, the frequency of the rater’s rating is unlikely to affect the overall mean rating scores.

### Development stage

***Objective 1: Face validity***. A preliminary version of the VASE ( Lai *et al.*, 2020b) was field-tested with six healthy adult volunteers from the general public. An opportunity sample was used where volunteers were approached by the researchers and invited to participate. Volunteers were recruited from the general public in Hong Kong, the Hub at the Wellcome Collection, Dementia Research Centre, University College London and Salomons Institute, Canterbury Christ Church University. The volunteers were asked to watch one of two YouTube videos preloaded onto the VASE app. Both video clips were around three minutes in duration and consisted of a musician delivering a music intervention to a person with advanced dementia.

Feedback was sought using open-ended questions on the usability of the app, and included the following: *"What are the particular behaviour/behaviours that made you feel that the person with dementia was engaging or not engaging?", "What are your thoughts about the current rating system?" and "What led to your decision in making your ratings?"*

*Qualitative data analysis.* Verbal feedback was incorporated into the earlier version of the measure and recorded in a questionnaire to further refine and revise the VASE. Using Braun & Clarke’s (2006) six-stage approach of thematic analysis, common patterns and categories about what engagement of people with dementia looks like were identified ( Table 1).

**Table 1.** Initial examples of categories and behavioural expressions of engagement.

	Category	Definition	Behaviour expression of engagement	Examples of the feedback (s)	No. of participants commenting on the categories
1	Facial expression	Noticeable changes on the PWD’s face during the intervention	*Mouth and lip movement*	“mouth moving, mumbling the song”	2
			*Eyebrows movement (e.g. closed their* *eyes or raise their eyebrow)*	“The person’s eyebrows were raising when the music was playing”	1
			*Facial changes (e.g. neutral look, smile)*	“I can see that the person (PWD) face looked different … like she was smiling) “The person face looked very neutral without much facial expression, but you can feel that she was enjoying the music, as if she was thinking about it.”	6
2	Bodily movement and verbal articulations	Large or small bodily movements and response during the intervention	*Large and subtitle bodily movement* *(e.g. hands and feet taping, nodding,* *clapping, moving with music)*	“Hand clapping and feet tapping” “There was one person who tapped his hands on his lap”	6
			*Verbally responding (e.g. singing,* *talking, mouth mumbling)*	“One of the elderly was moving her mouth.”	2
			*Interacting with instruments (touching* *the instrument, playing with the* *instruments, making music)*	“Hitting the African drum and the hand drum” “Playing with the drum stick”	3
3	Attention and awareness of activity	Being focus and attend to a stimulate that is in context with the intervention	*Attention to stimulus (musician, other* *participants) undistracted eye contact*	“there’s a lot of duplication. Because when they would said to you, “okay, this man has been accepted, could you please do a referral”, and we are all using information on the same system. So we end up doing the same thing again” “We’re having these discussions with psychology in the pod meeting they’re quite often quite like in-depth, which is great, but then if they’re accepted there and then in the meeting for psychology, the clinician, like the care coordinator then has to go away and type out the conversation. But we’ve already had the conversation with psychology, so could the referral not just be accepted there and then, without the paper part being done?”	4
			*Playing an instrument*	“Playing and focusing on the instruments in front of her”	
			*Moving along with music*	“Her feet was tapping (along with the music)”	
4	Emotional response	Participant’s “positive” and “negative” emotions in relations to the intervention	*Pleasure and enjoyment*	“I don’t really know what’s going on down here”	4
			*At ease look (looks as if s/he was* *relaxed)*	“She closed her eyes, but it looks like she was enjoying the music.” “Looking up but thinking about things but she seemed relaxed”	2
			*Sad or anxious look (appear agitated,* *e.g., eyes down casted like in moment* *of unhappiness; tapping his/her fingers* *as in people who are anxious)*	“I can see that the elderly was sad…but it does not mean she was not enjoying the music right? Maybe it made her remember something” “But I suppose negative emotions like looked anxious and sad can mean that the person is (PWD) is engaging with the therapy (intervention) right ?)”	2

PWD, person with dementia.

***Objective 1: Content validity***. Following open-ended feedback from the six volunteers, thematic analysis results were reviewed by four interdisciplinary experts (musician practicing music within residential care, neuropsychologist, clinical health psychologist and a trainee clinical psychologist), in order to revise and create a protocol that would more accurately reflect the rating of engagement in the VASE. After the protocol was completed and adjustments made the final version ( Lai *et al.*, 2020b) was further tested by two volunteers, from the Dementia Research Centre, University College London, who were verbally approached to participate.

### Experimental stage

The experimental stage was conducted in two parts. The first part involved recording active and passive music sessions (MfL and PL, respectively) involving people with advanced dementia living in a residential care home. The second part involved recruiting raters to rate brief video excerpts from the music sessions using the VASE. Each part of the study, the setting, participants and procedures, are described below.

**Part 1**

***Setting.*** Video recording of MfL and PL sessions were made in a London care home.

***Participants.*** Eight PWD participated. Recruitment criteria: (i) a confirmed diagnosis of dementia; (ii) Clinical Dementia Rating Scale ( Morris *et al.*, 1997) score of 2–3 (advanced) as rated by care home staff; (iii) aged 60 or above; and (iv) able to sit in a room for an hour in a group setting. PWD that had (i) a clinical dementia rating of below 2; (ii) significant hearing difficulties that cannot be corrected, even with a hearing aid; or (iii) disruptive behaviour during group activities in the care facility (e.g. aggressive behaviour) were excluded. These criteria were screened by care staff at the care home and verified by one of the researchers.

***The intervention (MfL) and passive listening (PL) conditions.*** The MfL programme is one type of music-based intervention used for advanced dementia. It is an interactive music programme that was designed to promote better quality of life for people with dementia in residential care ( Music for Life, 2014); it took place for one hour a week over a course of eight weeks. Each week specially trained musicians facilitate and attempt to establish and enhance communication with the PWD through improvisational music and activities. The PL session was held once, whereby participants listened to pre-recorded music that was similar to that used in the intervention sessions and played by the same musicians. The settings, day and time of day, length of session, number of musicians and care staff present at both intervention and control conditions were equivalent.

**Figure 1.**
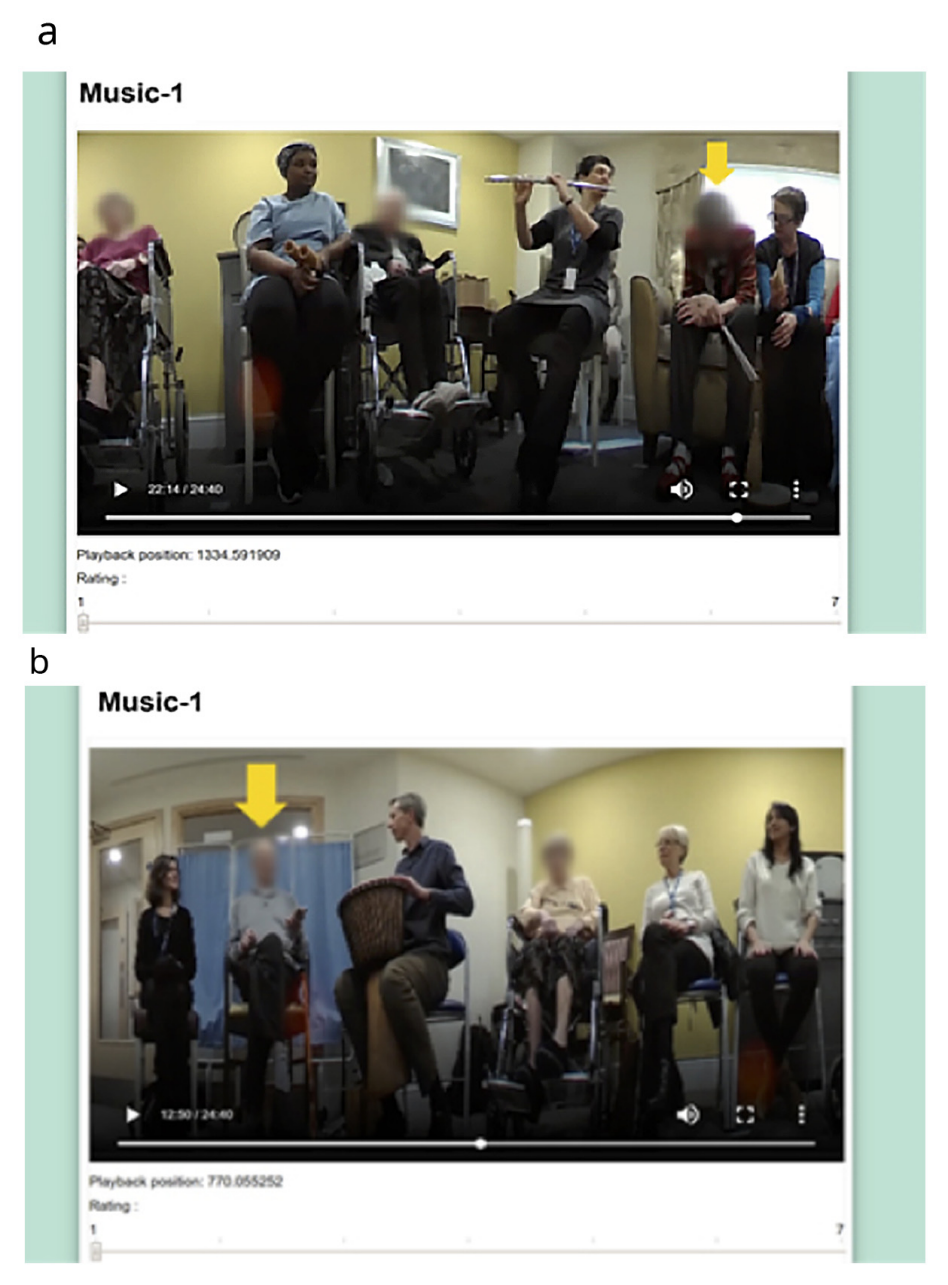
Snapshots of the final version of the VASE. We confirm that we have obtained permission to use images from the participants included in this presentation.

***Procedures.*** The experimental stage first involved a one-hour control session. This was followed one week later at the same time and in the same location by the start of the eight-week MfL intervention. At the beginning of each session, a 360-degree Fly video camera ® was placed in the middle of the room; this camera, which is smaller than a tennis ball, captures continuous 360-degree recording, making it ideal for use in groups. Videos were edited by an independent video editor into 30-second segments, and 12 from the control and 4–5 from each of the intervention sessions were chosen pseudo-randomly. In each segment one of the eight participants was randomly chosen for the raters to specifically focus on and this was indicated with a yellow arrow ( Figure 1). The 48 segments from the control and intervention sessions and PL session were then edited into a single 25-minute-long video. The order of the clips in the video was also pseudorandomised. Two more videos were made in the same manner as the first one, where the order of the clips was again pseudo-randomly assigned to remove potential order effects.

**Part 2**

***Setting.*** The video clips were then viewed using the tablet computers by the raters and scored in secure and non-public places. This included university and research organisations meeting rooms, a public library private meeting room and a charity’s office.

***Participants.*** Opportunity sampling was used to recruit professional and non-professional raters in Hong Kong and the UK through emails and face-to-face contact. Six professionals were included as raters (clinical and neuro psychologists, nurses and dementia charity managers). The inclusion criteria were as follows: (i) work in a health-related discipline; (ii) aged 18 or over; and (iii) one or more years of experience working in dementia care. Thirty-one people from the general public (non-professionals) aged 18 and above, who had not worked clinically with dementia, were also recruited.

***Sample size.*** In accordance with the Medical Research Council (MRC) framework (2000), this was a feasibility study. Lancaster, Dodd & Williamson (2004) recommend an overall sample size of 30 for feasibility studies. Table 2 presents the demographic characteristics of the raters.

**Table 2.** Demographic characteristics of raters.

	Total (N = 37)	
	N	Percent
Age (mean ± SD)	38.2 ± 2.69	
*Gender*		
Male	12	32
Female	25	68
*Education*		
High school or lower	2	5
Undergraduate	13	35
Master's	17	46
PhD or higher	5	14
*Ethnicity*		
White British	15	41
White other	4	11
Asian	17	46
Other	1	3
*Participation in singing group*		
Yes	23	62
No	14	38
*Currently in singing group*		
Yes	9	24
No	28	76
*Experience with playing musical instrument*		
Yes	17	46
No	20	54
*Experience with PWD*		
Yes	22	59
No	15	41
*Type of Raters*		
Professional	6	16
Non-professional	31	39

SD = standard deviation; PWD = people with dementia.

***Procedures.*** Once consent forms were signed, the raters were requested to provide demographic information and were given the protocol describing the categories of engagement to read. Raters were then given a password-protected tablet and over-ear headphones to complete a series of rating scales while watching the video recording of the MfL group. Research personnel were present to supervise this process and answer questions during all video viewings. Inter-rater reliability was tested to establish the consistency of the final version of the VASE.

**Data analysis**

Raw data from the VASE, including the time and ratings were recorded. The scores were then rounded to the nearest second. Data was then transferred into SPSS version 23 for analysis.

***Objective 2: Reliability.*** An inter-class correlation coefficient (ICC) was used to determine the consistency of coefficients across all raters. This was also used to assess the engagement scores across the three videos (different clip order: M1, M2 and M3), the conditions (PL and MfL session) and the raters (profession and non-professionals). Spearman’s correlation was used to evaluate the inter-correlations between the professionals, as well as between the non-professionals.

***Objective 3: Mixed model analysis.*** Multilevel linear modelling (MLM) was used to investigate the relationship between distribution of rating and average rating over time on raters’ characteristics. The analysis included the rating provided by raters overall, video conditions (MfL versus PL), gender, videos watched (different clip order: M1, M2 and M3), professional or non-professional, with or without experience in looking after PWD, and experience of musical instrument and singing group. Since one participant would appear in multiple clips and they were rated by each rater at these different time points, MLM takes account of the dependencies by estimating variance associated with group (e.g. raters) and differences in average response (intercepts). The model adopted for data analysis in this study regards intercepts and/or slopes to be random effects. All of the analyses were conducted using SPSS Version 23 and the level of the significance was set to 0.05.

### Ethical considerations

Ethics approval was granted by Canterbury Christ Church University, Salomons Institute Ethics Panel (approval number: V:\075\Ethics\2016-17) and also approved by the charity’s review panel (approval number: V1a28617) where the research was conducted. The study followed National Institute for Health Research guidelines for working with people who are unable to directly provide informed consent. An information sheet was given to caregivers and consent obtained from the participants’ primary caregiver, who had the legal authority to give consent. Before each video recording, centre staff and musicians would also verbally remind the participants that the sessions were being recorded for the purpose of this research and offer them the opportunity to withdraw from the recording; none withdrew. Raters were reminded they would be watching a recording of a vulnerable population and that they should not disclose the name or identifying information of people viewed in the video; all signed consent forms and agreed to abide by these requests. Video recordings were transferred from the camera directly to encrypted files in a password-protected tablet computer and the video data removed from the camera.

## Results

### Development stage

***Objective 1: Face validity.*** Based on the feedback from the volunteers watching the YouTube videos, some adjustments were made; this included adjusting the size of the videos to 850mm × 500mm and adjusting the font size. The volunteers reported finding it difficult to distinguish the different types of engagement. Some volunteers also expressed that watching the same video three times made them lose interest and, as a result, they found it difficult to concentrate during the repeat viewings. Consequently, it was decided that the VASE should be used to rate an overall state of engagement. After making the revisions on the app, a further field-test was carried out with five more volunteers to ensure that the final version of the VASE was suitable ( Figure 1). 

In addition, based on the questions related to aspects that made raters consider a PWD to be engaged in the group, a thematic analysis was carried out using the interview data gathered from volunteers. The interviews were transcribed and four main categories (patterns) and 12 behavioural expressions of the categories were finalised ( Table 3).

**Table 3.** Categories and behaviour expressions of engagement.

Categories	Behavioural expressions
Facial expressions	i. Mouth and lip movement
	ii. Eyebrows movement (e.g. closed their eyes or raise their eyebrow)
	iii. Facial changes (e.g. neutral look, smile)
Bodily movement and verbal articulations	i. Large and subtle bodily movement (e.g. Hands and feet taping, nodding, clapping, moving with music)
	ii. Verbally responding (e.g. singing, talking, mouth mumbling)
	iii. Interacting with instruments (touching the instrument, playing with the instruments, making music)
Attention and awareness of activity	i. Attention to stimulus (musician, other participants) undistracted eye contact
	ii. Playing an instrument
	iii. Moving along with music
Emotional response	i. Pleasure and enjoyment as indicated by smiles and a look of contentment
	ii. At ease look (looks as if s/he was relaxed)
	iii. Sad or anxious look (appear agitated, e.g., eyes down casted like in moment of unhappiness; tapping his/her fingers as in people who are anxious)

***Objective 1: Content validity.*** Four main categories were derived for the initial protocol based on the qualitative data analysis. The four categories included: a) facial expressions; b) bodily movement and verbal articulations; c) attention and awareness of activity; and d) emotional responses. The initial protocol was much briefer, offering little description of the categories. There was 100% agreement from the experts, indicating that the categories and their corresponding behavioural expressions are a good representation of engagement in people with advanced dementias. However, the experts also highlighted the importance of offering some examples of behaviours relating to each category. They specifically considered that explanations should include behavioural expressions that were not easily picked up as a sign of engagement. For instance, one of the experts spoke about PWD experiencing what other people might describe as “negative” emotions, such as sadness, where they might be considered to be emotionally “moved” by the music. The experts further commented that there is a need to highlight that sometimes eyes being closed, or even a neutral look, can be a sign of a person being engaged. In addition, the professional’s rating would also be dependent upon different cues, such as the context of the situation, the rater’s own experience, and the rater’s understanding of the group. Consequently, some experts proposed that the protocol should not be a rigid manual; rather, it should simply provide a reference for what engagement is and allow a certain amount of ambiguity and openness towards a rater’s own interpretation. The appropriateness of the rating would be determined in the third stage of inter-rater reliability testing. To see if there was consistency and agreement, different raters’ scoring of the same individual at the same time were statistically tested to examine if variances existed. Consequently, a statement about there being no right or wrong answer was added to the brief description. This process resulted in the final version of the VASE protocol.

### Experimental stage

**Figure 2.**
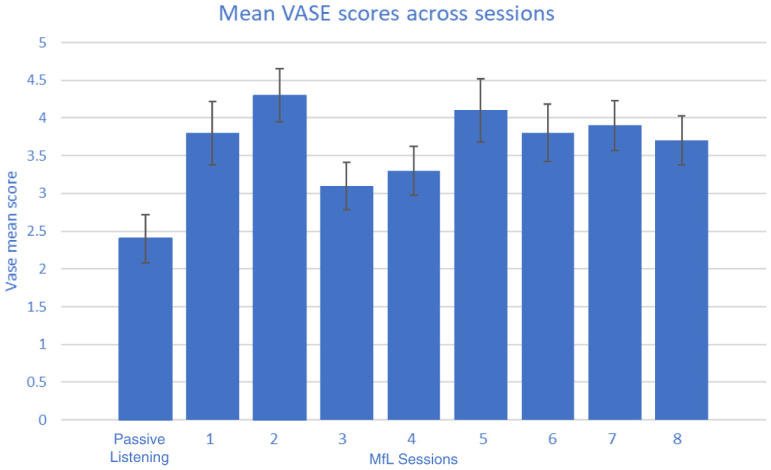
Mean (standard error) Video Analysis Scale of Engagement (VASE) scores across all sessions.

Observing raters during the viewing sessions, and from informal comments made by the raters, it was apparent that they had a variable delay in making their rating as each new video appeared and they appraised the scenario. As a result, the first five seconds of each clip were excluded from the analysis of the rating data gathered. In total, each rater watched 1,200 seconds of clips, of which 300 seconds were PL and 900 seconds were MfL sessions. The VASE was tested across 37 raters. The mean rating scores of each individual rater under MfL and PL conditions are reported in Table 5. A non-parametric Mann-Whitney U test was used to analyse whether there were any differences in rating between the two conditions by each rater. The results showed that most of the raters (36 out of 37 raters) gave significantly higher ratings for MfL than PL sessions. These higher ratings were observed irrespective of the order in which the videos were viewed and rated (M1, M2 or M3) or their professional/non-professional status ( Table 4). It should be noted that the mean MfL ratings shown in Table 4 and Table 5 are mean scores of MfL sessions 1 to 8 ( Figure 2). Differences between MfL and PL mean ratings suggest that the inter-class correlation should be analysed separately for MfL and PL sessions.

**Table 4.** Scoring by individual rater as analysed by MfL and PL conditions.

		Total			PL			MfL			Mann-Whitney U test
Raters	Video	Mean	SD	Median	Mean	SD	Median	Mean	SD	Median	
1	M1	2.4	1.41	2.0	1.7	0.78	2.0	2.7	1.49	2.0	10.752 ***
2	M1	4.1	2.18	4.0	4.5	2.38	5.0	3.9	2.09	4.0	4.297 ***
3	M1	3.9	2.03	4.0	2.7	1.91	2.0	4.3	1.92	4.0	11.211 ***
4	M1	3.2	1.56	3.0	2.4	1.50	2.0	3.5	1.48	3.0	10.605 ***
5	M1	3.8	2.24	3.0	2.2	1.55	2.0	4.3	2.19	4.0	14.089 ***
6	M1	4.1	2.33	4.0	2.8	2.14	2.0	4.5	2.23	4.0	11.298 ***
7	M1	3.0	1.62	3.0	2.1	1.22	2.0	3.3	1.61	3.0	12.340 ***
8	M1	1.8	1.23	1.0	1.2	0.44	1.0	2.1	1.32	1.0	11.539 ***
9	M1	4.3	2.08	5.0	2.7	1.64	2.0	4.8	1.93	5.0	15.319 ***
10	M1	4.5	1.99	5.0	3.0	1.33	3.0	5.1	1.91	6.0	15.564 ***
11	M2	3.7	1.91	4.0	2.4	1.64	2.0	4.2	1.78	4.0	14.244 ***
12	M2	3.1	1.33	3.0	2.3	1.19	2.0	3.3	1.27	3.0	11.143 ***
13	M2	1.9	1.40	1.0	1.3	0.71	1.0	2.1	1.51	1.0	8.628 ***
14	M2	1.6	0.88	1.0	1.3	0.57	1.0	1.7	0.94	1.0	6.692 ***
15	M2	4.5	1.97	5.0	3.2	1.88	3.0	4.9	1.80	5.0	12.806 ***
15	M2	5.5	2.12	7.0	4.7	2.46	6.0	5.8	1.93	7.0	5.942 ***
17	M2	2.8	1.78	2.0	1.8	1.08	1.0	3.1	1.85	3.0	11.570 ***
18	M2	3.8	2.16	4.0	2.6	2.03	1.0	4.2	2.06	4.0	11.314 ***
19	M2	5.1	1.68	6.0	4.0	1.86	5.0	5.5	1.43	6.0	12.870 ***
20	M2	2.6	1.84	2.0	2.7	2.08	2.0	2.6	1.75	2.0	0.962
21	M2	3.1	1.61	3.0	1.8	1.03	1.0	3.5	1.52	3.0	17.696 ***
22	M2	2.7	2.16	1.0	1.7	1.70	1.0	3.0	2.19	2.0	11.201 ***
23	M3	2.4	1.56	2.0	2.1	1.17	2.0	2.5	1.65	2.0	2.928 ***
24	M3	3.4	1.88	4.0	2.3	1.49	2.0	3.8	1.85	4.0	12.061 ***
25	M3	2.9	1.16	3.0	2.1	0.89	2.0	3.1	1.12	3.0	13.274 ***
26	M3	2.4	1.60	2.0	1.7	0.85	1.0	2.7	1.72	2.0	8.709 ***
27	M3	3.4	1.79	3.0	2.2	1.14	2.0	3.8	1.79	4.0	13.809 ***
28	M3	3.2	2.07	2.0	1.8	1.08	1.0	3.7	2.09	4.0	14.341 ***
29	M3	3.9	2.02	4.0	2.1	1.27	1.0	4.5	1.84	5.0	18.253 ***
30	M3	4.4	1.77	5.0	3.1	1.72	3.0	4.9	1.53	5.0	14.695 ***
31	M3	1.9	1.23	1.0	1.6	0.93	1.0	1.9	1.30	1.0	3.200 ***
Ratings by professionals											
A	M1	4.0	1.92	4.0	2.8	2.00	2.0	4.4	1.72	4.0	11.732 ***
B	M1	4.2	1.74	5.0	2.8	1.57	3.0	4.7	1.52	5.0	16.059 ***
C	M2	4.6	2.18	5.0	3.4	2.21	3.0	5.0	2.02	6.0	10.239 ***
D	M3	3.6	1.68	4.0	2.4	1.40	2.0	4.1	1.55	4.0	14.502 ***
E	M3	3.6	1.73	4.0	2.2	1.29	2.0	4.1	1.57	4.0	16.928 ***
F	M3	3.0	1.56	3.0	1.9	0.84	2.0	3.3	1.58	3.0	14.404 ***

*** p < 0.005. SD = standard deviation; MfL = Music for Life; PL = passive music listening; M1, M2, M3 = video order.

**Table 5.** Influence of video rating order and professional status upon engagement ratings for MfL and PL conditions.

	PL			MfL			Mann- Whitney U test
Video (order)	Mean	SD	Median	Mean	SD	Median	
M1	2.6	1.14	2.5	4.0	1.29	3.8	14.636 ***
M2	2.5	1.05	2.4	3.8	1.21	3.8	14.243 ***
M3	2.1	0.83	2.1	3.5	1.17	3.6	17.382 ***
Professionals	2.6	1.25	2.5	4.3	1.15	4.2	17.443 ***
Non- professionals	2.4	0.94	2.2	3.7	1.10	3.7	15.413 ***

*** p < 0.005. SD = standard deviation; MfL = Music for Life; PL = passive music listening; M1, M2, M3 = video order.

### Objective 2: Reliability

***Inter-rater reliability.*** In our study, the ICC was used to measure the consistency of raters in the rating of the engagement of the target participants over 48 video clips. According to Koo and Li’s criteria (2016), values less than 0.5 are considered to have poor reliability, 0.5 to 0.75 are considered to have moderate reliability, 0.75 to 0.9 is considered to have good reliability and ICCs greater than 0.9 are considered to have excellent reliability.

The ratings of the three videos (different clip order) of M1, M2 and M3 and the ratings for MfL and PL sessions were assessed for their inter-rater reliability. The values of ICC in the MfL sessions ranged between 0.841 and 0.876, while the corresponding values in the PL session ranged between 0.812 and 0.883. All of the values were greater than 0.8, which is considered to reflect good reliability. Comparing the ratings of professional experts and the general public, the values of ICC in both groups indicated an excellent level of reliability (MfL: 0.881; PL: 0.938). These results help accept hypothesis 1 and indicate that the professional experts and the general public showed strong agreement in both conditions (MfL and PL). The mean ICC values suggest the reliability levels were good among the professionals (MfL: 0.854; PL: 0.775) and excellent among the non-professionals (MfL: 0.918; PL: 0.916), although the different group sizes (professional: N = 6; non-professional: N = 31) should be noted in interpreting these values. The findings accept hypothesis 2 and suggest that there is a correlation between the VASE rating among the dementia experts, as well as among non-professionals.

The mean rating scores of the raters across videos (M1, M2 and M3) were assessed ( Table 6). For M1 and M2, the values of ICC were greater than 0.9 for both M1 and M2, indicating excellent reliability. The values of ICC for M1 and M3, as well as M2 and M3, showed good reliability with some achieving moderate reliability. 

**Table 6.** Intraclass correlation coefficient (ICC) *.

	Value	95% CI
*Inter-rater reliability*		
M1	0.896	(0.869, 0.916)
MfL	0.876	(0.839, 0.903)
PL	0.867	(0.823, 0.898)
M2	0.861	(0.804 , 0.897)
MfL	0.841	(0.770, 0.885)
PL	0.812	(0.747, 0.858)
M3	0.901	(0.876, 0.920)
MfL	0.869	(0.828, 0.898)
PL	0.883	(0.858, 0.905)
Professional vs non-professional	0.920	(0.637, 0.967)
MfL	0.881	(0.258, 0.957)
PL	0.938	(0.909, 0.956)
Professional	0.850	(0.811, 0.878)
MfL	0.854	(0.812, 0.886)
PL	0.775	(0.717, 0.818)
Non-professional	0.934	(0.920, 0.944)
MfL	0.918	(0.898, 0.935)
PL	0.916	(0.897, 0.931)
Inter-retest reliability (across groups)		
M1 vs M2	0.951	(0.939, 0.960)
MfL	0.936	(0.913, 0.951)
PL	0.959	(0.948, 0.967)
M1 vs M3	0.804	(0.713, 0.859)
MfL	0.710	(0.617, 0.774)
PL	0.867	(0.498, 0.943)
M2 vs M3	0.786	(0.743, 0.820)
MfL	0.679	(0.630, 0.722)
PL	0.866	(0.523, 0.941)

* All of ICC values are significant with p < 0.001. CI = confidence interval; MfL = Music for Life; PL = passive music listening; M1, M2, M3 = video order.

Spearman’s correlation coefficients between the professionals were evaluated. The results show that the coefficients ranged from 0.353 to 0.72 ( Table 7). In our study, there was a high correlation between experts E and F, which indicated that they had a high level of agreement in their ratings. The correlation coefficients among experts B, E and F were greater than 0.5, which indicates that they had a moderate correlation. The correlation between experts D, E and F, as well as that between experts A, B and C was also moderate. On the other hand, experts A and C had a weak correlation with D, E, and F. All the correlation coefficients indicated a statistically significant degree of correlation. Correlation coefficients between the non-professionals were also calculated, with results showing that 89.5% (416 out of 465) of the correlation coefficients between raters are significantly correlated at 5% level ( Table 8). In general, the findings support hypothesis 2, indicating that there is a correlation between the VASE rating among professionals, as well as non-professionals.

**Table 7.** Spearman’s correlation coefficient among professionals.

Raters	A	B	C	D	E	F
A	1					
B	0.641 ***	1				
C	0.535 ***	0.644**	1			
D	0.371 ***	0.403 ***	0.402 ***	1		
E	0.441 ***	0.599 ***	0.466 ***	0.575 ***	1	
F	0.353**	0.571 ***	0.396**	0.500 ***	0.720 ***	1

*** p < 0.005.

**Table 8.** Spearman’s correlation coefficient among non-professionals.

	1	2	3	4	5	6	7	8	9	10	11	12	13	14	15	16
A1_M1 (1)	1.000															
A5_M1 (2)	0.097 **	1.000														
A8_M1 (3)	0.564 **	0.033	1.000													
AS4_M1 (4)	0.647 **	0.016	0.717 **	1.000												
CCIL_3_M1 (5)	0.803 **	0.107 **	0.683 **	0.711 **	1.000											
CCIL_5_M1 (6)	0.715 **	0.111 **	0.632 **	0.673 **	0.819 **	1.000										
CCIL_7_M1 (7)	0.662 **	0.126 **	0.629 **	0.580 **	0.768 **	0.724 **	1.000									
DRC5_M1 (8)	0.668 **	-0.030	0.565 **	0.584 **	0.685 **	0.591 **	0.647 **	1.000								
DRC8_M1 (9)	0.546 **	0.042	0.478 **	0.472 **	0.687 **	0.591 **	0.656 **	0.636 **	1.000							
DRC9_M1 (10)	0.565 **	0.021	0.559 **	0.625 **	0.651 **	0.521 **	0.550 **	0.629 **	0.563 **	1.000						
A6_M2 (11)	0.691 **	0.031	0.720 **	0.599 **	0.762 **	0.772 **	0.738 **	0.593 **	0.602 **	0.594 **	1.000					
A9_M2 (12)	0.620 **	-0.015	0.616 **	0.621 **	0.698 **	0.654 **	0.701 **	0.605 **	0.591 **	0.602 **	0.697 **	1.000				
AS3_M2 (13)	0.683 **	0.137 **	0.549 **	0.517 **	0.714 **	0.567 **	0.663 **	0.695 **	0.605 **	0.531 **	0.581 **	0.587 **	1.000			
AS6_M2 (14)	0.493 **	0.030	0.362 **	0.388 **	0.513 **	0.513 **	0.489 **	0.456 **	0.453 **	0.425 **	0.458 **	0.422 **	0.467 **	1.000		
CCIL_1_M2 (15)	0.591 **	0.059 *	0.583 **	0.525 **	0.701 **	0.624 **	0.732 **	0.607 **	0.689 **	0.547 **	0.735 **	0.669 **	0.565 **	0.359 **	1.000	
CCIL_4_M2 (16)	0.392 **	0.024	0.330 **	0.263 **	0.371 **	0.434 **	0.432 **	0.279 **	0.284 **	0.378 **	0.534 **	0.456 **	0.262 **	0.258 **	0.407 **	1.000
CCIL_6_M2 (17)	0.706 **	0.110 **	0.617 **	0.582 **	0.794 **	0.648 **	0.755 **	0.636 **	0.624 **	0.497 **	0.645 **	0.577 **	0.662 **	0.428 **	0.647 **	0.235 **
CCIL_8_M2 (18)	0.653 **	0.198 **	0.601 **	0.506 **	0.731 **	0.654 **	0.712 **	0.600 **	0.632 **	0.589 **	0.688 **	0.625 **	0.658 **	0.479 **	0.666 **	0.476 **
DRC1_M2 (19)	0.490 **	0.016	0.493 **	0.422 **	0.620 **	0.554 **	0.613 **	0.568 **	0.585 **	0.653 **	0.667 **	0.622 **	0.493 **	0.378 **	0.592 **	0.441 **
DRC2_M2 (20)	-0.035	0.052	0.099 **	0.075 **	-0.009	-0.076 **	0.013	0.043	0.084 **	0.072 *	0.095 **	0.173 **	0.003	-0.035	0.154 **	-0.003
DRC4_M2 (21)	0.599 **	-0.026	0.631 **	0.579 **	0.718 **	0.673 **	0.713 **	0.569 **	0.673 **	0.566 **	0.762 **	0.708 **	0.541 **	0.448 **	0.740 **	0.415 **
DRC7_M2 (22)	0.553 **	-0.041	0.556 **	0.506 **	0.651 **	0.557 **	0.677 **	0.598 **	0.625 **	0.603 **	0.613 **	0.604 **	0.591 **	0.512 **	0.659 **	0.303 **
A4_M3 (23)	0.060 *	-0.012	0.080 **	-0.008	0.059 *	0.044	0.146 **	0.077 **	0.030	-0.076 **	0.092 **	-0.012	0.082 **	0.024	0.047	0.059 *
A7_M3 (24)	0.617 **	-0.008	0.566 **	0.581 **	0.661 **	0.628 **	0.651 **	0.523 **	0.490 **	0.444 **	0.595 **	0.592 **	0.548 **	0.338 **	0.546 **	0.248 **
AS5_M3 (25)	0.501 **	-0.077 **	0.511 **	0.521 **	0.547 **	0.489 **	0.493 **	0.491 **	0.473 **	0.393 **	0.437 **	0.376 **	0.544 **	0.332 **	0.424 **	0.162 **
CCIL_2_M3 (26)	0.575 **	-0.025	0.519 **	0.582 **	0.628 **	0.554 **	0.533 **	0.528 **	0.486 **	0.420 **	0.546 **	0.464 **	0.484 **	0.302 **	0.455 **	0.170 **
DRC10_M3 (27)	0.482 **	-0.004	0.382 **	0.434 **	0.530 **	0.514 **	0.586 **	0.490 **	0.496 **	0.430 **	0.470 **	0.461 **	0.427 **	0.300 **	0.501 **	0.221 **
DRC11_M3 (28)	0.610 **	-0.031	0.554 **	0.595 **	0.666 **	0.612 **	0.662 **	0.579 **	0.500 **	0.456 **	0.610 **	0.558 **	0.526 **	0.345 **	0.543 **	0.299 **
DRC3_M3 (29)	0.475 **	-0.118 **	0.491 **	0.561 **	0.623 **	0.519 **	0.597 **	0.524 **	0.520 **	0.519 **	0.561 **	0.553 **	0.430 **	0.312 **	0.587 **	0.305 **
DRC6_M3 (30)	0.083 **	-0.023	0.047	-0.005	0.097 **	0.083 **	0.157 **	0.083 **	0.138 **	0.000	0.170 **	0.012	0.115 **	0.132 **	0.131 **	0.159 **
AS3_M3 (31)	0.591 **	-0.115 **	0.530 **	0.560 **	0.637 **	0.573 **	0.621 **	0.624 **	0.555 **	0.504 **	0.609 **	0.549 **	0.537 **	0.374 **	0.611 **	0.319 **

**Table:** 

Spearman’s correlation coefficient among non-professionals (Cont’d)															
	17	18	19	20	21	22	23	24	25	26	27	28	29	30	31
A1_M1 (1)															
A5_M1 (2)															
A8_M1 (3)															
AS4_M1 (4)															
CCIL_3_M1 (5)															
CCIL_5_M1 (6)															
CCIL_7_M1 (7)															
DRC5_M1 (8)															
DRC8_M1 (9)															
DRC9_M1 (10)															
A6_M2 (11)															
A9_M2 (12)															
AS3_M2 (13)															
AS6_M2 (14)															
CCIL_1_M2 (15)															
CCIL_4_M2 (16)															
CCIL_6_M2 (17)	1.000														
CCIL_8_M2 (18)	0.606 **	1.000													
DRC1_M2 (19)	0.466 **	0.585 **	1.000												
DRC2_M2 (20)	0.026	-0.085 **	0.176 **	1.000											
DRC4_M2 (21)	0.587 **	0.631 **	0.661 **	0.116 **	1.000										
DRC7_M2 (22)	0.551 **	0.666 **	0.549 **	0.011	0.739 **	1.000									
A4_M3 (23)	0.141 **	0.123 **	0.078 **	-0.017	0.068 *	-0.002	1.000								
A7_M3 (24)	0.600 **	0.538 **	0.396 **	-0.138 **	0.549 **	0.488 **	0.047	1.000							
AS5_M3 (25)	0.468 **	0.413 **	0.362 **	-0.065 *	0.437 **	0.443 **	0.014	0.667 **	1.000						
CCIL_2_M3 (26)	0.520 **	0.459 **	0.371 **	-0.068 *	0.455 **	0.344 **	0.171 **	0.776 **	0.659 **	1.000					
DRC10_M3 (27)	0.484 **	0.506 **	0.422 **	-0.053	0.490 **	0.484 **	0.002	0.710 **	0.656 **	0.627 **	1.000				
DRC11_M3 (28)	0.567 **	0.539 **	0.443 **	-0.098 **	0.592 **	0.488 **	0.090 **	0.821 **	0.643 **	0.787 **	0.732 **	1.000			
DRC3_M3 (29)	0.522 **	0.509 **	0.438 **	0.045	0.623 **	0.573 **	0.048	0.678 **	0.557 **	0.643 **	0.695 **	0.724 **	1.000		
DRC6_M3 (30)	0.091 **	0.084 **	0.076 **	-0.037	0.056	0.061 *	0.564 **	0.120 **	0.155 **	0.174 **	0.052	0.093 **	0.086 **	1.000	
AS3_M3 (31)	0.546 **	0.521 **	0.496 **	-0.003	0.614 **	0.550 **	0.036	0.731 **	0.669 **	0.714 **	0.688 **	0.739 **	0.702 **	0.098 **	1.000

** p < 0.01; * p<0.05.

### Objective 3: Mixed model analysis

**Figure 3.**
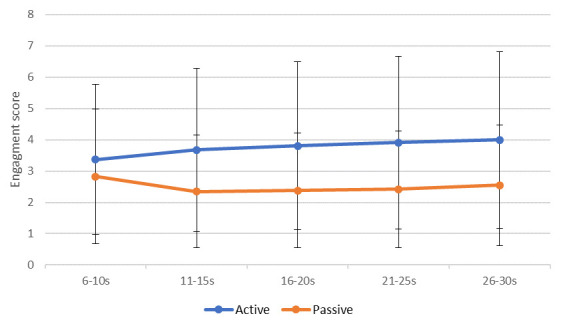
Mean (standard error) engagement ratings between Music for Life and passive listening sessions across the five periods.

Data were averaged into five five-second periods and recoded into new variables called “sequence”. This variable categorised each five seconds of a clip into periods: period 1 (6s–10s), period 2 (11s–15s), period 3 (16s–20s), period 4 (21s–25s) and period 5 (26–30s) ( Figure 3).

MLM was used to investigate the relationship between the rated level of engagement and a number of variables, including condition type (active [MfL]/passive [PL]), session number (1–8), within-video “sequence” (1–5), and rater characteristics, such as age, gender, professional status (professional/non-professional), experience of playing an instrument, experience of singing in a choir, presence in one of the MfL sessions, and experience of PWD (all were dichotomous ‘yes/no’ ratings).

In our hypothetical model, MfL or PL condition, sessions, engagement across the five “period”, raters’ profile such as age, gender, profession, playing an instrument and experience of singing in a choir, and experience with dementia were entered as fixed effects (without interaction term), while rater and PWD recorded in the videos were entered as random effects, which is based on the hypothesis that there would be a difference in the relationship between the level of engagement and raters, as well as the PWDs. 

A full model (-2 log likelihood = 167030.821) that includes all of the variables is significantly better than one in which only the intercepts are included (-2 log likelihood = 173760.602), with λ ^2^ (10, N = 44400) = 6729.781, p < 0.001. Thus, inclusion of all variables improved the model beyond that produced by considering variability in raters and participants. This significantly lower level of chi-square in the full model justified the use of MLM. Among the 10 predictors selected for the model, half of them were significantly associated with the level of engagement ( Table 9). These five variables were period (β = 0.12, p < 0.005), MfL or PL condition (β = -1.43, p < 0.005), session (β = 0.02, p < 0.005), age (β = -0.03, p < 0.005) and profession (β = 0.97, p < 0.05).

**Table 9.** Estimates of fixed effects ^a^

						95% Confidence interval	
Parameter	Estimate	Std. Error	df	t	Sig.	Lower bound	Upper bound
Intercept	4.844	0.501	37.203	9.672	0.000	3.830	5.859
Period	0.122	0.005	44101.510	23.105	0.000	0.111	0.132
Condition	-1.435	0.026	44353.786	-54.713	0.000	-1.486	-1.383
Session	0.024	0.004	44201.130	5.350	0.000	0.015	0.033
Age	-0.031	0.009	37.000	-3.319	0.002	-0.050	-0.012
Profession	0.970	0.382	37.000	2.541	0.015	0.196	1.743
Rater gender	-0.073	0.264	37.000	-0.276	0.784	-0.608	0.462
Instrument	-0.349	0.360	37.000	-0.970	0.338	-1.079	0.380
Presently in sing group	0.746	0.372	37.000	2.006	0.052	-0.007	1.500
Sing group	-0.470	0.309	37.000	-1.519	0.137	-1.097	0.157
PWDexp	-0.614	0.384	37.000	-1.600	0.118	-1.391	0.163

^a^ Dependent variable: rating.

Level of engagement was significantly associated with five of the factors in the model ( Table 9):

1. Sequence (β = 0.12, p < 0.0): ratings differed significantly across the five “period”, and on average went up by 0.12 per period, indicating that raters’ ratings were changing over time.
2. MfL or PL condition (β = -1.43, p < 0.005): Engagement was rated significantly higher for the MfL sessions than the PL session, on average 1.43 points on the rating scale.
3. Significant differences across sessions (β = 0.02, p < 0.005): Ratings were recorded as higher at the latest session than the earlier session, with on average 0.02 points difference per session, which might not be clinically significant.
4. Age of rater (β = -0.03 p < 0.005): Younger raters provided higher ratings than their older counterparts.
5. Professional raters (β = 0.970, p < 0.05): Professional raters provided significantly higher ratings.

Table 10 shows the random effects of the model. It was found that there was significant variability in the ratings given by different raters (p < 0.001), as well as significant variability in the rating of the PWD between raters (p < 0.001). There was also significant residual variance after taking into account all effects in the model. This residual variance might indicate that the model requires more variables. The residuals of the model were tested with a Q-Q plot: it was found that the residuals followed a normal distribution and it was thus concluded that the normality assumption of the model is supported.

**Table 10.** Estimates of covariance parameters ^a^

						95% Confidence interval	
Parameter		Estimate	Std. error	Wald Z	Sig.	Lower bound	Upper bound
Residual		2.456	0.017	148.495	0.000	2.424	2.489
Intercept [subject = Rater *VidSubject]	Variance	0.679	0.062	10.939	0.000	0.567	0.812
Intercept [subject = Rater]	Variance	0.424	0.119	3.556	0.000	0.245	0.736

^a^ Dependent variable: rating.

## Discussion

The aim of this feasibility and validation study was to develop an easily accessible and user-friendly engagement measure for use in the assessment of engagement of people with advanced dementia. The study helped better understand engagement in people with advanced dementia by creating an operational definition of the concept. It also showed the possibility of adopting VASE as a measure for the aforementioned use, particularly in group settings. It also demonstrates the potential utility of continuous, multi-rating scales that capture dynamic changes in a rateable concept like engagement from moment-to-moment. In the study, face validity was obtained based on the opinions of volunteers from the general public. Thematic analysis of interview data was used to construct an operational definition of engagement. Inter-rater-reliability was documented, and strong agreement was found in some conditions.

Hypothesis 1 testing the correlation between VASE rating by dementia professionals and non-professional people is accepted. ICC indicated that VASE has good to excellent agreement between the two samples of professionals and general public. Yet, the MLM suggested that overall, professionals generally provided significantly higher rating values than the general public (average of 0.97 Likert scale points). This could be a result of professionals having more clinical knowledge and understanding about engagement of people with advanced dementia than the general public, greater awareness of the challenges PWD experience in their capacity to respond verbally and physically, or altered perceptions of the significance of subtle behaviours.

Hypothesis 2 tested inter-rater reliabilities and is accepted. When looking at inter-rater reliability across professionals, high to moderate ICC were found in general with some inconsistent results between raters. This inconsistency might be the result of experts working in different fields and different settings. For example, those who had higher agreement with each other were psychologists and clinicians working in community settings who have experience running groups for PWD. The others work in more acute hospital settings; consequently, they have less experience of residential group interventions. As a result, the professionals working in acute wards might potentially have different understandings of what engagement looks like for PWD than those working in residential care. Indeed, a person’s belief system, worldview and reality are often constructed based on their experience ( Koltko-Rivera, 2004). This was difficult to account for and capture in the VASE and it raises questions about whether the current VASE is overly reductionist in capturing such a complex concept as engagement. On the other hand, high agreement was found from non-professionals, further supporting the reliability of VASE among non-professional raters.

 Hypothesis 3 tested whether VASE will be able to differentiate the level of engagement between MfL and PL, and is accepted. The ICC and non-parametric testing results suggested that the VASE has good to excellent reliability in differentiating MfL from PL sessions. These results echo the findings from MLM where raters generally rated the control condition an average of 1.44 Likert scale points lower. This result is perhaps unsurprising as active MfL music activities are more dynamic and interactive than passive music listing, with participants tending to react and respond to each other and the musician(s) also making the music using different musical instruments.

 Hypothesis 4 assumed that the extraneous variables would not affect rating. Hypothesis 4 is rejected. The MLM findings suggest that ratings differ significantly across periods (five seconds). This suggests that the level of engagement rated in the video changes over time and raters are using the VASE in a dynamic fashion. Another promising result was that the order in which raters rated the segments (M1 versus M2 versus M3) had no effect on rating value. This is consistent with the between-group ICC result and suggests that the VASE rating scale is not affected by order effects.

The rater’s age seemed to have an effect on the rating score. During data collection some participants aged in their 70s and above expressed that they found it difficult to use the tablets and a 25-minute video was too long for them. Cornish & Dukette (2009) stated that the average maximum attention time for adults is around 20 minutes, therefore, future research should consider the optimal length of time to use the VASE. Apart from familiarity with technology and attention span, other factors such as decline in processing speed ( Eckert *et al.*, 2010) and response selection time ( Woods *et al.*, 2015), have been found to be associated with ageing. To overcome this issue, it is worth considering using tests for reaction time commonly used in computerised neurocognitive tests to learn about raters’ baseline reaction times ( Schatz *et al.*, 2015), or revise the program in such a way that permits a longer processing duration or to pause and rewind.

### Strength and weaknesses

The present study developed a new rating scale and examined its validity. This study explored the possibility of using “non-symptomatic” concepts such as engagement to understand PWD’s response in interventions. Engagement with others and involvement in activities are important for various dimensions of health and wellbeing for those with dementia ( Benveniste *et al.*, 2012; Robinson *et al.*, 2013). A validated scale for the assessment of engagement will be useful to researchers and clinicians to better understand the effect of interventions for PWD and those who might have difficulties verbally expressing themselves. The examination of the processes during an intervention is crucial in helping professional and family carers to learn about the participants’ responses, and to gauge clinical benefits. VASE is therefore a useful measure in that sense. When such a measure is user-friendly and publicly available, it will have wider applicability to all those who are involved in dementia care.

Secondly, the study made a unique contribution as it was the first known study to capture moment-by-moment changes of engagement that take place during the intervention, enabling raters to continuously make ratings as they observe changes in the video. The VASE is also non-intrusive and it does not require raters to be present due to the use of a previously recorded video. Videos can be reviewed and re-rated again by the same viewer or different viewers, enabling multiple raters to cross-track their engagement scores. Most importantly, the VASE can record the exact time that changes in engagement occur during a group intervention. This allows raters to know which particular activities stimulate different levels of engagement for particular individuals. This could potentially enable clinicians or carers to tailor specific activities for PWD in order to promote person-centred care. This scale might also allow family members who are living at a distance to be involved in suggesting activities for their family member via a live link or through pre-recorded video.

Moreover, the engagement scale was complemented by the decision to use a 360-degree camera to record group sessions. This permitted a simultaneous and much wider view in identifying engagement and interaction between participants in a group than single point of view recordings or direct observation by a single observer present at the session.

One of the limitations of VASE is that this was a feasibility study and the sample size of raters was relatively small. Furthermore, as sampling was opportunistic, there is the possibility of sampling bias ( Stasser & Titus, 1985), and people who participated in this experiment might have different attitudes and understandings about engagement from those who did not participate in the study.

Criterion and construct validity were also not established. It was not possible to establish criterion validity as the VASE is a single item continuous rating scale. However further research could determine construct validity to enable better confidence that VASE is operating theoretically as expected ( Gaugler *et al.*, 2013). Test-retest reliability was also not established due to funding and time constraints. Further, rating participants on two music group conditions (PL and MfL) does not ensure the results are generalisable to others.

Lastly, during the discussion on the use of VASE, some experts proposed that in order to be able to fully understand engagement in a session, it is necessary to be present in the group and be “immersed in that atmosphere”, yet others disagreed with this. Some experts also commented that ratings of engagement might be subjective and based on the raters’ understanding of, and familiarity with, the subject area they are rating. As a result, there might be factors that this study had not considered. As this is the first cycle in its development, further work is needed before we can be confident in its reliability.

### Research implications

Future research needs to review the current version of VASE and investigate possible adjustments of the measure, such as trialling with ratings based on different types of engagement and reviewing the duration of the rated video segments. Further research should also investigate the difference between family members and professionals in their understanding of engagement of PWD living in care homes. The protocol could then be revised to consider these differences. With a larger sample size different validity and reliability tests could be used, such as criterion and construct validity. Test-retest reliability could also be considered with the same rater re-rating the video.

The VASE adopted a seven-point Likert rating; seven-point ratings have been previously recommended as a good multi-point scale in preference to a five-point scale ( Lewis, 1993). However, the results of the mixed model suggest that the mean difference (standard estimate) between conditions, such as session and conditions (control and intervention) was small; further examination on the sensitivity and specificity of the VASE is needed to better understand the statistical and clinical significance of outcomes.

In the MLM, residual (unexplained) variance reached a significant level. This suggests there are currently other variables that have not been considered and more variables need to be incorporated in order to build a better model ( Heck & Thomas, 2000). Variables such as mood, cognitive ability, attitude towards the activity could be added. Raters’ awareness of dementia and attitudes towards dementia ( Handley *et al.*, 2017) and their age and baseline reaction time could also be investigated.

Further research could examine the use of VASE in other interventions regarded as beneficial for PWD such as cognitive stimulation ( Orrell *et al.*, 2017), art therapy ( Deshmukh *et al.*, 2018) and other types of music interventions ( Clare & Camic, 2020).

### Dementia care implications

The mixed findings suggest that further refinement of the VASE is needed before it can be used in dementia care settings. Engagement as an outcome was not previously considered a worthwhile construct to measure in a dominant medical model of dementia care but it is now deemed valuable with person-centred ( Kitwood, 1997b; Sung & Chang, 2005) and relational ( Greenwood *et al.*, 2001) approaches. Unlike symptom-based tools that measure the success of the intervention the VASE offers an alternative way of understanding dementia, investigating choice and interaction. With revision, the VASE could be adopted in residential care settings to help understand levels of engagement with various different activities. This could enable care staff to assess whether particular activities are suitable for individuals. As VASE provides a time stamp in terms of noted changes in engagement, it could also assist group facilitators to identify particular activities or stimuli that support higher levels of engagement and those that support less. This could enable facilitators to adjust their intervention based on the group or the individual’s preference. Facilitators could also take the measurement and results to other experts, and even clinical supervision, in order to make changes that would benefit PWD.

As a video analysis measurement that is easy to use and user-friendly, the VASE could potentially be beneficial for inviting a wider support network to engage in the care of the individual. For example, it could enable family carers to view and evaluate activities in which PWD participate in a care home without needing to be present in the group, allowing families the option of becoming more involved in care.

Lastly, if the measure is found to be valid, it could potentially be used for staff training, where examples of engagement and non-engagement can be identified and shared. Most importantly, the new observational tool could also enable us to gain a better understanding of the particular nuances and components of what makes an activity useful for this population, and potentially this could be applied to evaluations of other types of interventions and activities (e.g. museum object handling, approaches to self-care, family interactions). Dynamic rating scales beyond engagement could also be adopted, applying them to other concepts in dementia care where observation of dynamic changes is required.

## Conclusion

The feasibility and validation study results indicate that the current version of the VASE has good reliability in some areas. It still needs further investigation and adjustments for it to be a valid and reliable tool in measuring engagement of people with dementia in a group setting within residential care. Balancing the wish to develop a user-friendly measurement tool and to capture a complex abstract concept such as engagement is challenging. It is encouraging that there is some evidence suggesting that the VASE is able to distinguish between the level of engagement of participants in two different types of music activities (passive and active). With further adjustments and investigation, the VASE could be a useful measure in advancing dementia care. Improvements in assessments of the processes during an intervention will facilitate a better capturing of the concept of engagement, and would eventually benefit carers in the promotion of wellbeing of those who are affected by dementia.

## Data availability

### Underlying data

The raw data analysed in this research are based on a video analysis from a multi-session music group for people with advanced dementia. Family members who are legal guardians for the participants in this study provided written consent for their participation as did the residential care facility where data was collected. Due to the sensitive nature of video data showing full frontal identification we are not able to make the videos available in an open access format. Permission to do so was not given by the ethics review panels, as well as being inappropriate to do for a vulnerable group of research participants.

If researchers are interested in using the video data from this study they are asked to contact the corresponding author ( p.camic@ucl.ac.uk) describing the nature of their interest, the intended use of the data, plans for obtaining ethical approval at their respective institution and signing a confidentiality agreement to assure protection of data whilst in their possession. When ethical approval has been provided as evidenced by a signed letter from the ethics panel, data will be transferred via overnight carrier in an encrypted external hard drive at the requester’s expense. Once received, video data may not be downloaded onto other computers but can only be accessed from the external password protected and encrypted hard drive, which must be returned at the end of its use to the corresponding author.

Zenodo: Video Analysis Scale of Engagement project: data set. https://doi.org/10.5281/zenodo.4001112 ( Lai *et al.*, 2020a).

This project contains the following underlying data in the file ‘Mfl_data.csv’:

- Validity testing data
- Rating data

### Extended data

Zenodo Repository: Video Analysis Scale of Engagement (VASE): Initial and Final Protocols. https://doi.org/10.5281/zenodo.4001099 ( Lai *et al.*, 2020b).

This project contains the following extended data:

- VASE Initial Protocol.docx
- VASE Final Protocol.docx

Data are available under the terms of the Creative Commons Attribution 4.0 International license (CC-BY 4.0).

## Software availability

Source code available from: https://github.com/PaulCamic/Video-Analysis-Scale-of-Engagement-VASE-for-people-with-advanced-dementia/tree/VASE-source-code

Archived source code at time of publication: https://doi.org/10.5281/zenodo.4001025 ( Lai *et al.*, 2020c)

License: Creative Commons Zero v1.0 Universal
