# Supplementary material for: Development of the Video Analysis Scale of Engagement (VASE) for people with advanced dementia
Source: Wellcome Open Res. 2021 Jun 18;5:230. Originally published 2020 Oct 5. [Version 3] doi: 10.12688/wellcomeopenres.16189.3 (PMC8311801; doi:10.12688/wellcomeopenres.16189.3)
Supplement: Peer review history

# Peer review: Reviewer response for version 3

**DOI:** 10.21956/wellcomeopenres.18759.r44570

**Published:** 2021-07-22

**Authors:** Sara Isernia

**Affiliations:** 1 IRCCS Fondazione don Carlo Gnocchi ONLUS, Milan, Italy

I appreciated the effort of the authors in addressing my concerns. I believe that the manuscript is now suitable to be accepted for indexing.

Is the work clearly and accurately presented and does it cite the current literature?

Yes

If applicable, is the statistical analysis and its interpretation appropriate?

Partly

Are all the source data underlying the results available to ensure full reproducibility?

Yes

Is the study design appropriate and is the work technically sound?

Yes

Are the conclusions drawn adequately supported by the results?

Yes

Are sufficient details of methods and analysis provided to allow replication by others?

Partly

Reviewer Expertise:

NA

I confirm that I have read this submission and believe that I have an appropriate level of expertise to confirm that it is of an acceptable scientific standard.

# Peer review: Reviewer response for version 2

**DOI:** 10.21956/wellcomeopenres.18657.r43984

**Published:** 2021-06-07

**Authors:** Hiroko H Dodge

**Affiliations:** 1 Department of Neurology, Oregon Health & Science University, Oregon, USA

The authors addressed my previous concerns.

 One minor comment I would like to add: is it possible to report the number of time-stamped responses for each rater in Table 4? This information might add another interesting aspect of this measurement (e.g., professional raters rate more frequently compared with non-professional raters).

Is the work clearly and accurately presented and does it cite the current literature?

Yes

If applicable, is the statistical analysis and its interpretation appropriate?

Yes

Are all the source data underlying the results available to ensure full reproducibility?

Yes

Is the study design appropriate and is the work technically sound?

Yes

Are the conclusions drawn adequately supported by the results?

Yes

Are sufficient details of methods and analysis provided to allow replication by others?

Partly

Reviewer Expertise:

Gerontology, Dementia, Behavioral Intervention, Neurology, Biostatistics.

I confirm that I have read this submission and believe that I have an appropriate level of expertise to confirm that it is of an acceptable scientific standard.

# Peer review: Reviewer response for version 1

**DOI:** 10.21956/wellcomeopenres.17774.r43360

**Published:** 2021-04-21

**Authors:** Hiroko H Dodge

**Affiliations:** 1 Department of Neurology, Oregon Health & Science University, Oregon, USA

This is a well written paper, examining validities of new tools to measure levels of engagement among the demented - an important field of research which currently lacks reliable objective measurements. The authors paid attention to methods in detail and it is easy to follow. I only request a minor clarification. 

 Raters score participants’ engagement levels in real-time while watching videos and each rating is time-stamped. It is not clear how frequently raters rate the engagement levels (e.g., every 30 seconds, or whenever raters believe engagement levels changed)? Is there variability in terms of frequency when raters give ratings? If there is, how does it affect (or not affect) the overall mean rating scores?

 Rating time is time-stamped. Can the time information be utilized for measuring rater differences? For example, is it possible to see the rater differences when ratings were provided at the exact same moment?

Is the work clearly and accurately presented and does it cite the current literature?

Yes

If applicable, is the statistical analysis and its interpretation appropriate?

Yes

Are all the source data underlying the results available to ensure full reproducibility?

Yes

Is the study design appropriate and is the work technically sound?

Yes

Are the conclusions drawn adequately supported by the results?

Yes

Are sufficient details of methods and analysis provided to allow replication by others?

Partly

Reviewer Expertise:

Gerontology, Dementia, Behavioral Intervention, Neurology, Biostatistics.

I confirm that I have read this submission and believe that I have an appropriate level of expertise to confirm that it is of an acceptable scientific standard, however I have significant reservations, as outlined above.

# Response

**Published:** 2021-06-04

**Authors:** Paul Camic

**Affiliations:** University College London, UK

**In response to Reviewer 2 (Dr Hiroko H Dodge)**

 Thank you for your helpful comments. Our responses are below.

**1. **   Raters score participants’ engagement levels in real-time while watching videos and each rating is time-stamped. It is not clear how frequently raters rate the engagement levels (e.g., every 30 seconds, or whenever raters believe engagement levels changed)? Is there variability in terms of frequency when raters give ratings? If there is, how does it affect (or not affect) the overall mean rating scores?

**Response:** Thank you for your question. As mentioned in the Methods session, p. 33, 2nd paragraph titled, Video analysis scale of engagement (VASE) the “VASE adopts a continuous scoring system using a seven-point Likert-type scale to assess the level of engagement whilst viewing a video segment. Responses and time of the responses are automatically recorded by the software.” Therefore, the frequency of rating was dependent upon the individual rater’s decision and judgement. In our study, we captured the rater’s rating to the nearest second. Each second of score was captured whether the rater has changed the score or the score has stayed the same. All participants would, therefore, have a total of 1200 seconds of score, which is the total length of the video. Thus, the frequency of the rater’s rating is unlikely to affect the overall mean rating scores. We have added an explanation in the text for further clarity. 

**2.  **   Rating time is time-stamped. Can the time information be utilized for measuring rater differences? For example, is it possible to see the rater differences when ratings were provided at the exact same moment?

**Response**: As each rater have a total of 1200 seconds of score, we have been able to look at the raters difference in the nearest second. In our study, for example, high agreement was found from non-professional rater’s, where 89.5% of the correlation coefficients between raters are significantly correlated at the 5% level.  

 Thank you.

 Daniel Lai, Sebastian Crutch and Paul Camic (in behalf of all authors)

# Peer review: Reviewer response for version 1

**DOI:** 10.21956/wellcomeopenres.17774.r40713

**Published:** 2020-10-21

**Authors:** Sara Isernia

**Affiliations:** 1 IRCCS Fondazione don Carlo Gnocchi ONLUS, Milan, Italy

The present manuscript reports an interesting and relevant study on the validity and reliability of a new tablet computer-based observational measure to assess engagement in dementia. I found the study technically sound and appropriate.

 I suggest some clarifications and modifications:

1. When authors reported Objective 2 they referred to intra-rater reliability. However, they run a comparison between different raters (even if within professionals’ groups). They would have assessed intra-rater reliability whether they correlated the assessment of engagement of the same rater in different moments.
2. To evaluate face validity of the preliminary version of the VASE, authors referred to open-ended questions on the usability of the app. I suggest reporting in the manuscript or in the supplementary materials the usability questions.
3. A detailed statistical analysis section is lacking. Data analysis section includes only minor information. I suggest integrating this part by linking each analysis to the objectives of the study.

Is the work clearly and accurately presented and does it cite the current literature?

Yes

If applicable, is the statistical analysis and its interpretation appropriate?

Partly

Are all the source data underlying the results available to ensure full reproducibility?

Yes

Is the study design appropriate and is the work technically sound?

Yes

Are the conclusions drawn adequately supported by the results?

Yes

Are sufficient details of methods and analysis provided to allow replication by others?

Partly

Reviewer Expertise:

NA

I confirm that I have read this submission and believe that I have an appropriate level of expertise to confirm that it is of an acceptable scientific standard, however I have significant reservations, as outlined above.

# Response

**Published:** 2020-11-05

**Authors:** Paul Camic

**Affiliations:** University College London, UK

Dear Dr Isernia,

 Thank you for your review of our articles and the comments you made. Much appreciated. Regarding your first comment, “When authors reported Objective 2 they referred to intra-rater reliability. However, they run a comparison between different raters (even if within professionals’ groups). They would have assessed intra-rater reliability whether they correlated the assessment of engagement of the same rater in different moments.”, apologies but we are not clear on what you would like us to alter? We ran both intra and inter reliability checks;

 From page 8, “An intra-class correlation coefficient (ICC) was used to determine the consistency of coefficients across all raters. This was also used to assess the engagement scores across the three videos (different clip order: M1, M2 and M3), the conditions (PL and MfL session) and the raters (profession and non-professionals). Spearman’s correlation was used to evaluate the inter-correlations between the professionals, as well as between the non-professionals.”

 Regarding your second comment, “To evaluate face validity of the preliminary version of the VASE, authors referred to open-ended questions on the usability of the app. I suggest reporting in the manuscript or in the supplementary materials the usability questions”, yes, we will add these within the manuscript of the revised manuscript when we have received comments from the second reviewer.

 And finally, in your third comment you state, “A detailed statistical analysis section is lacking. Data analysis section includes only minor information. I suggest integrating this part by linking each analysis to the objectives of the study.” We have reported our statistical analyses as we have done them and it is not clear to us what is meant by “only minor information” is included. Apologies, but we are not sure what is lacking? Would you like us to add additional tables and figures as extended data?

 Thank you for your further assistance.

 The authors,

 Daniel Lai, Sebastian Crutch and Paul Camic

# Response

**Published:** 2021-06-04

**Authors:** Paul Camic

**Affiliations:** University College London, UK

**Responding to Reviewer 1 (Dr. Sara Isernia)**

 Thank you for your helpful comments. We have added additional information as requested below. We very much appreciate your input into our work.

 1.    From the authors’ comment (to my earlier review) I guessed they adopted the ICC to assess intra-rater reliability. My concern was related to the fact that, whether I correctly understood, participants rated patients using VASE once. In this sense, you have a measure of the variation between 2 or more raters who measure the same group of subjects (inter-rater reliability) and not a measure of the variation of data measured by 1 rater across 2 or more trials (intra-rater reliability). Did authors utilize an adequate form of ICC to assess intra-rater reliability

 Response: Thank you for your clarification, the word intra-rater reliability has now been removed. This was an unintended mistake on our part. We used ICC to assess inter-rater reliability, or the consistency of raters’ ratings across people, events, or tasks (Shrout & Fleiss, 1979). There are six different types of calculation of ICC. We used ICC (2,k), which implies that each participant is rated by each rater, and raters are considered representative of a large population of similar raters. The reliability is calculated by taking an average of the k raters’ measurement.

 2.    Regarding the data analysis section, I found not complete this part because it is not linked to the separate objectives of the study. I believe that linking each analysis to the related aim could ameliorate the clarity of the manuscript. Also, no mention of the qualitative analysis is reported in this section

 Response: Thank you for your comment, the objectives are now added in the analysis section and results section. Regarding qualitative analysis in the data analysis section, the title of data analysis has now been added in the development stage under the face validity section.

 3.    To evaluate face validity of the preliminary version of the VASE, authors referred to open ended questions on the usability of the app. I suggest reporting in the manuscript or in the supplementary materials the usability questions.

 Response: We have added the following questions to the text: "What are the particular behaviour/behaviours that made you feel that the person with dementia was engaging or not engaging?", "What are your thoughts about the current rating system?" and "What led to your decision in making your ratings?"

 Thank you.

 Daniel Lai, Sebastian Crutch and Paul Camic (on behalf of all authors)
